# Are Racial and Ethnic Minorities Less Willing to Participate in Health Research?

**DOI:** 10.1371/journal.pmed.0030019

**Published:** 2005-12-06

**Authors:** David Wendler, Raynard Kington, Jennifer Madans, Gretchen Van Wye, Heidi Christ-Schmidt, Laura A Pratt, Otis W Brawley, Cary P Gross, Ezekiel Emanuel

**Affiliations:** **1**Department of Clinical Bioethics, National Institutes of Health Clinical Center, National Institutes of Health, Bethesda, Maryland, United States of America; **2**Office of Behavioral and Social Sciences Research, National Institutes of Health, Bethesda, Maryland, United States of America; **3**National Center for Health Statistics, Centers for Disease Control and Prevention, Hyattsville, Maryland, United States of America; **4** Department of Epidemiology, Yale University School of Medicine, New Haven, Connecticut, United States of America; **5**Statistics Collaborative, Washington, D. C., United States of America; **6**Winship Cancer Institute, Emory University, Atlanta, Georgia, United States of America; **7**Section of General Internal Medicine, Yale University School of Medicine, New Haven, Connecticut, United States of America; University of BirminghamUnited Kingdom

## Abstract

**Background:**

It is widely claimed that racial and ethnic minorities, especially in the US, are less willing than non-minority individuals to participate in health research. Yet, there is a paucity of empirical data to substantiate this claim.

**Methods and Findings:**

We performed a comprehensive literature search to identify all published health research studies that report consent rates by race or ethnicity. We found 20 health research studies that reported consent rates by race or ethnicity. These 20 studies reported the enrollment decisions of over 70,000 individuals for a broad range of research, from interviews to drug treatment to surgical trials. Eighteen of the twenty studies were single-site studies conducted exclusively in the US or multi-site studies where the majority of sites (i.e., at least 2/3) were in the US. Of the remaining two studies, the Concorde study was conducted at 74 sites in the United Kingdom, Ireland, and France, while the Delta study was conducted at 152 sites in Europe and 23 sites in Australia and New Zealand. For the three interview or non-intervention studies, African-Americans had a nonsignificantly lower overall consent rate than non-Hispanic whites (82.2% versus 83.5%; odds ratio [OR] = 0.92; 95% confidence interval [CI] 0.84–1.02). For these same three studies, Hispanics had a nonsignificantly higher overall consent rate than non-Hispanic whites (86.1% versus 83.5%; OR = 1.37; 95% CI 0.94–1.98). For the ten clinical intervention studies, African-Americans' overall consent rate was nonsignificantly higher than that of non-Hispanic whites (45.3% versus 41.8%; OR = 1.06; 95% CI 0.78–1.45). For these same ten studies, Hispanics had a statistically significant higher overall consent rate than non-Hispanic whites (55.9% versus 41.8%; OR = 1.33; 95% CI 1.08–1.65). For the seven surgery trials, which report all minority groups together, minorities as a group had a nonsignificantly higher overall consent rate than non-Hispanic whites (65.8% versus 47.8%; OR = 1.26; 95% CI 0.89–1.77). Given the preponderance of US sites, the vast majority of these individuals from minority groups were African-Americans or Hispanics from the US.

**Conclusions:**

We found very small differences in the willingness of minorities, most of whom were African-Americans and Hispanics in the US, to participate in health research compared to non-Hispanic whites. These findings, based on the research enrollment decisions of over 70,000 individuals, the vast majority from the US, suggest that racial and ethnic minorities in the US are as willing as non-Hispanic whites to participate in health research. Hence, efforts to increase minority participation in health research should focus on ensuring access to health research for all groups, rather than changing minority attitudes.

## Abstract 2

Minorities in the US are as willing as non-Hispanic whites to participate in health research. Efforts to increase minority participation should focus on ensuring equal access to health research rather than changing minority attitudes.

## Introduction

**Figure 1:**
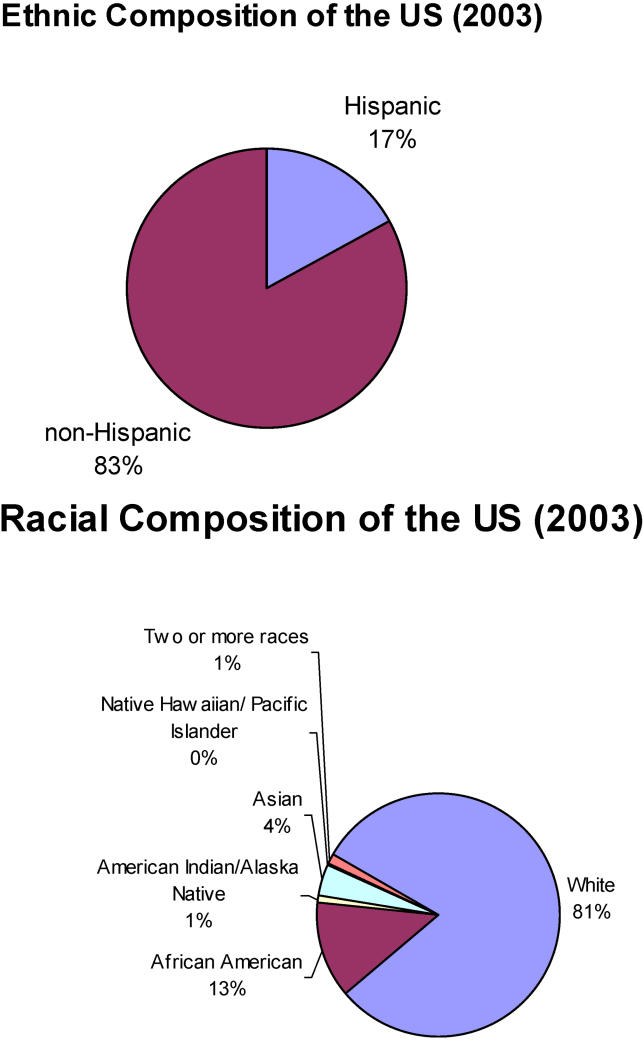
Ethnic and Racial Composition of the United States Data from year 2003.

To ensure the generalizability of research results, it is important that all groups participate in health research [1–4]. However, many commentators claim that racial and ethnic minority groups, especially in the US, are less willing to participate in health research [5–13]. While the US population includes an increasing percentage of individuals from minority groups, non-Hispanic whites still compose a majority of the population (Figure 1). It is widely believed that racial and ethnic minority groups in the US, especially African-Americans, are less willing than non-Hispanic whites to participate in health research. Many commentators believe that this relative unwillingness traces to past abuses, especially the notorious Tuskegee Syphilis Study [14–18], described as “the singular reason behind African-American distrust of the institutions of medicine and public health” [19].

The claim that racial and ethnic minority groups in the US are less willing to participate in health research seems to be validated by data showing that minority groups are underrepresented in at least some health research studies [20–25]. Yet, willingness to participate is just one factor that influences whether individual patients and patient groups participate in health research [9,26]. Other factors include whether they are informed of research opportunities, whether they are medically eligible to participate, and whether their personal circumstances, including child care demands, job flexibility, and geographic proximity to research sites, allow them to participate.

Simply assuming that minority groups' underrepresentation in some health studies is a result of their being less willing to participate may focus efforts aimed at increasing their participation on changing minority attitudes. If, however, minorities are equally willing to participate, and their lower participation in some studies traces to other factors, these efforts may prove ineffective, or even counterproductive. The assumption that minority groups are less willing to participate in health research also may inadvertently increase stigmatization, suggesting that minority groups are unwilling to bear their fair share of the burdens required to improve medical care.

A few studies have assessed the willingness of racial and ethnic minority groups to participate in individual research trials and trials that focus on single diseases [7,27,28]. However, we could find no published empirical data on the actual consent rates of minority groups for health research in general. To evaluate the widespread claim that racial and ethnic minorities are less willing to participate in health research, we assessed whether individuals from minority groups who were eligible and invited to participate in health research consented to enroll less frequently than non-Hispanic whites.

## Methods

### Non-Intervention Studies

The Tuskegee Syphilis Study, thought to be a major reason, particularly in the US, behind racial and ethnic minority groups' presumed unwillingness to participate in health research, was a US government-funded, epidemiologic study. To assess racial and ethnic minority groups' willingness to participate in current US government-funded, epidemiologic studies, we evaluated the health surveys conducted by the US National Center for Health Statistics (NCHS) for the most recent year for which data were available, year 2000.

The NCHS, the nation's principal health statistics agency, conducts two ongoing population based health surveys—the National Health Interview Survey (NHIS) and the National Health and Nutrition Examination Survey (NHANES). The NCHS, in collaboration with the National Immunization Program, also conducts the National Immunization Survey (NIS), which collects data that can be used to determine consent rates by race. The other health surveys conducted by the NCHS did not qualify for analysis, either because they are based on the NHIS sample, or because they are based on administrative records, not direct contact with individuals. Thus, three survey or non-intervention studies conducted by the NCHS that provide data on consent rates by race or ethnicity are included in the analysis.

The NHIS is an annual, in-person, household interview, designed to produce data representative of the civilian, non-institutionalized population of the US [29]. The sample consists of approximately 106,000 persons, in approximately 43,000 households in over 300 primary sampling units. The first part of the survey collects basic demographic and health data on all members of the household. A sample adult and child are then selected from each household to complete a more detailed interview that assesses illness, health-care utilization, and socioeconomic and demographic factors. All households selected into the sample are visited by interviewers to introduce the study and conduct a brief interview to determine eligibility. Data on race and ethnicity are collected at this initial contact. The NHIS invites a higher percentage of African-Americans and Hispanics to participate than the percentage of these two groups in the US population. Interview response rates were calculated for sample adults for whom demographic information was obtained during the initial contact. The NHIS neither conducts public outreach nor provides financial incentives.

The NIS is an annual, random-digit-dialing telephone survey of approximately 34,000 US households with at least one child 19–35 mo old [30]. After answering questions about the resident child's immunization status, participants are asked for approval to contact providers to obtain the child's immnunization records. The NIS neither conducts public outreach nor provides financial incentives.

The NHANES is an annual nationally representative sample survey of approximately 5,000 non-institutionalized US civilians. Extensive media and public outreach are conducted in each community to familiarize potential participants with the survey. The household interview component of the NHANES assesses respondents' health, health-care utilization, and demographic characteristics [31]. Participants do not receive any financial incentives for the interview portion of the NHANES.

Individuals who complete the interview portion of the NHANES are invited to participate in an extensive medical examination, which requires a separate consent. The medical examination lasts approximately 4 h and includes a physical examination and a second interview. The physical examination collects blood and urine samples for laboratory tests such as cholesterol levels, blood lead levels, and levels of exposure to other environmental health hazards. The interview includes questions on physical health, mental health, sexual behavior, and drug use. Participants who complete the physical examination receive $70–$100 for their time and effort. Because individuals must have already consented to the NHANES interview to be invited to participate in the medical examination, the consent rates for the medical examination are listed (Table 1), but not included in the overall statistical analysis.

**Table 1:**
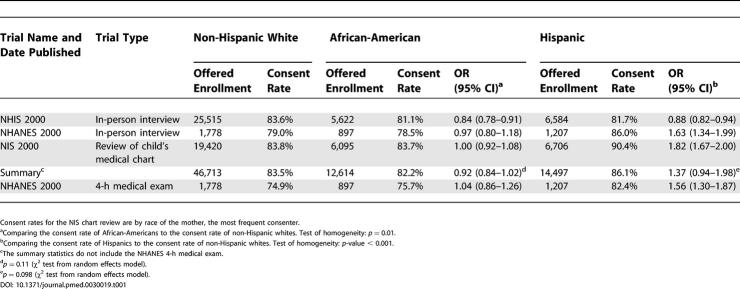
Interviews and Non-Intervention Studies

### Health Intervention Studies

Because there are no databases of health intervention trials, we conducted a comprehensive literature search of published trials. Using PubMed (http://www.ncbi.nlm.nih.gov/entrez/), we searched 30 unique combinations of terms and strings of terms related to enrollment, refusal, race, and ethnicity in phase I trials, phase I/II trials, phase II trials, and randomized controlled trials (see Table S1 for search terms). Studies were eligible for inclusion if they documented the race or ethnicity of eligible individuals invited to enroll, as well as the race or ethnicity of those who actually enrolled.

Two authors (G. V. and C. P. G.) reviewed the titles of all 1,681 articles identified by the search terms, then retrieved abstracts for the 1,106 articles that included any terms related to consent rates, including “consent,” “eligible,” “refusal,” or “enrollment” (see Figure S1 for a description of the selection process). The abstracts of all 1,106 retrieved articles were reviewed for any terms or phrases suggesting that they might include data by race or ethnicity, including “race,” “ethnicity,” “Caucasian,” “African-American,” “Hispanic,” and “non-Hispanic white.” The full texts of the 68 articles that included any of these terms were reviewed to determine whether they included data on consent rates by race or ethnicity, yielding 17 unique articles.

Next, Web of Science was used to search for authors whose names appeared in the citations of two or more of the 17 identified articles, yielding 371 articles (see Table S2). Using the same selection process, the bibliographies of the 17 identified articles were reviewed for any articles mentioning consent or participation rates, yielding another 89 articles. The same two authors (G. V. and C. P. G.) repeated the selection process to search the original 1,681 articles to determine whether any of these additional 467 articles included data on consent rates by race or ethnicity. This search yielded no additional articles.

Finally, to assess whether our search missed any studies that document consent rates by race or ethnicity, we evaluated the articles published for an entire year for two different types of trials. First, we reviewed all randomized controlled trials published during the 1-y period beginning April 1, 1999, in four major clinical journals: *Annals of Internal Medicine, JAMA, The Lancet,* and *The New England Journal of Medicine.* Because we wanted to assess individual patients' willingness to enroll in health research, trials were included only if they used individual patients as the unit of randomization (as opposed to hospital, region, etc.). This search identified 172 articles. We next conducted a MedLine search to identify all phase I oncology trials that used safety as an endpoint, published in English in the year 2002. This search identified 250 articles.

Using the same search process that was used for the original 1,681 studies, all 422 so identified articles were reviewed to determine whether any documented consent rates by race or ethnicity. This search yielded one study that documented consent rates by race, which had been identified previously by our original MedLine search. The fact that this search of the published articles for an entire year for two different types of intervention trials did not yield any new articles suggests that our original search likely identified all studies published in English that documented consent rates by race or ethnicity.

### Statistical Analysis

We defined the consent rates for a given study as the number of individuals in each reported racial or ethnic group who agreed to participate in the study divided by the number of individuals in that group who were invited to participate. The identified studies classified minority groups in four different ways: (1) African-Americans and Hispanics classified separately; (2) only African-Americans classified; 3) African-Americans classified separately and all other minorities grouped together; 4) all minorities grouped together. Because the original studies used different race/ethnic classifications, the minority group(s) to which non-Hispanic whites are compared in the present analysis varies across the studies.

For each study, we calculated an odds ratio (OR) and the associated 95% confidence interval (CI) using non-Hispanic whites as the reference group. The OR specifies, for each study, whether the reported minority group was more or less likely to consent to enrollment than non-Hispanic whites. An OR greater than one indicates that the minority group was more likely to consent than non-Hispanic whites; an OR of less than one indicates that the minority group was less likely to consent. A DerSimonian–Laird random effects model was used to estimate the summary OR and 95% CI for each type of study (i.e., non-intervention studies, clinical intervention studies, and surgical intervention studies), again using non-Hispanic whites as the reference group [32].

We considered the summary OR to be statistically significant if the 95% CI did not include one. We also used the following statistic to test for statistical significance:



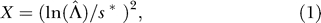



where 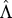 is the estimated summary OR and *s* ^*^ is the estimated standard deviation of the log summary OR. Under the null hypothesis that Λ = 1, *X* has a χ^2^ distribution with one degree of freedom. We also tested for homogeneity by calculating a Breslow–Day χ^2^ statistic.

## Results

We found 20 health research studies that included sufficient data to determine consent rates by race or ethnicity. Eighteen of the twenty studies were single-site studies conducted exclusively in the US or multi-site studies where the majority of sites (i.e., at least 2/3) were in the US. Of the remaining two studies, the Concorde study was conducted at 74 sites in the United Kingdom, Ireland, and France, while the Delta study was conducted at 152 sites in Europe and 23 sites in Australia and New Zealand. Taken together, these 20 studies reported the enrollment decisions of over 70,000 individuals, the vast majority of whom were from the US, for a broad range of health research studies, from interviews and non-intervention studies to drug treatment and surgical trials.

**Figure 2:**
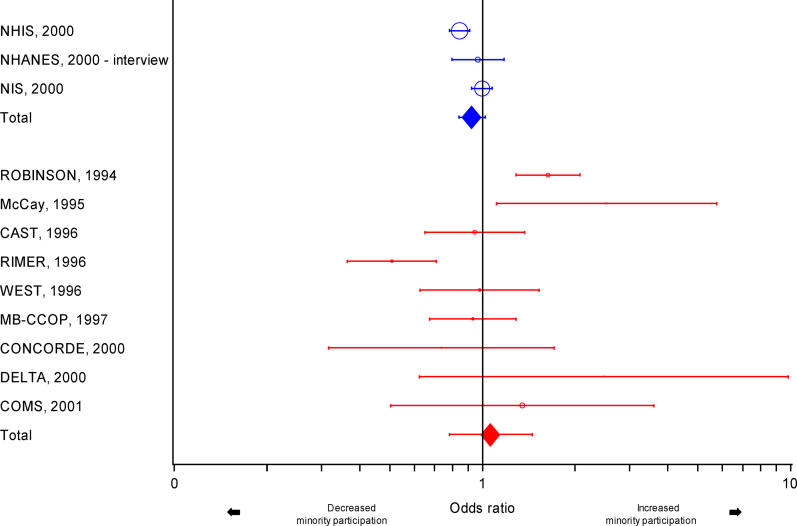
Comparison of African-American versus non-Hispanic White Consent Rates Circle diameter is proportional to the sample size of the individual studies. The diamond represents the overall OR. The vertical line indicates the 95% confidence interval on the OR. Blue indicates interview and non-intervention studies; red indicates clinical intervention studies.

**Figure 3:**
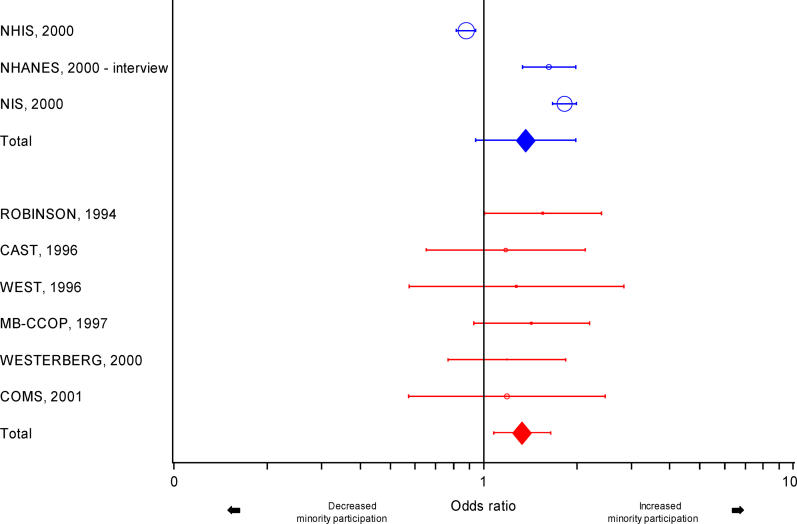
Comparison of Hispanic versus non-Hispanic White Consent Rates Circle diameter is proportional to the sample size of the individual studies. The diamond represents the overall OR. The vertical line indicates the 95% confidence interval on the OR. Blue indicates interview and non-intervention studies; red indicates clinical intervention studies.

For the three interview or non-intervention studies, African-Americans had a nonsignificantly lower overall consent rate than non-Hispanic whites (82.2% versus 83.5%; OR = 0.92; 95% CI 0.84–1.02; Table 1; Figure 2). For these same three studies, Hispanics had a nonsignificantly higher overall consent rate than non-Hispanic whites (86.1% versus 83.5%; OR = 1.37; 95% CI 0.94–1.98; Figure 3). Additionally, there was a significant lack of homogeneity for both comparisons (i.e., the test of homogeneity of the OR was rejected). A lack of homogeneity indicates that the relative willingness to enroll of minority groups versus non-Hispanic whites was not consistent, but varied significantly within the group of studies.

For the ten clinical intervention studies, African-Americans had a nonsignificantly higher overall consent rate than non-Hispanic whites (45.3% versus 41.8%; OR = 1.06; 95% CI 0.78–1.45; Table 2; Figure 2). Again, there was a significant lack of homogeneity among the ORs, indicating that the relative willingness to enroll of minority groups versus non-Hispanic whites varied significantly within the group of studies. For these same ten studies, Hispanics had a statistically significant higher overall consent rate than non-Hispanic whites (55.9% versus 41.8%; OR = 1.33; 95% CI 1.08–1.65; Figure 3).

**Table 2:**
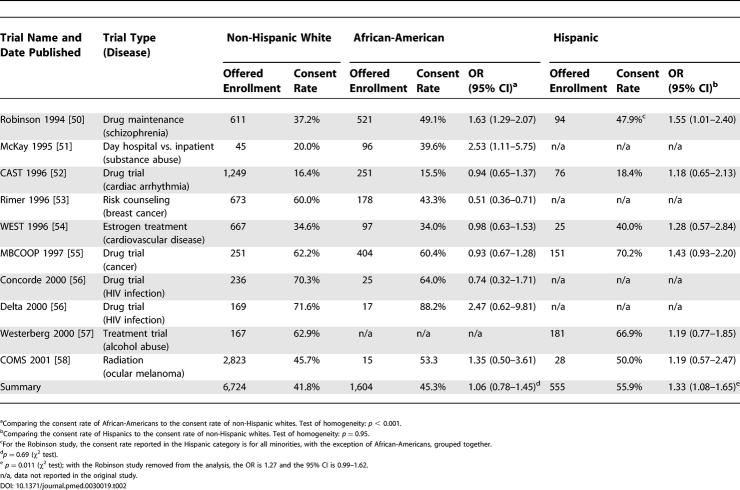
Clinical Intervention Trials

**Figure 4:**
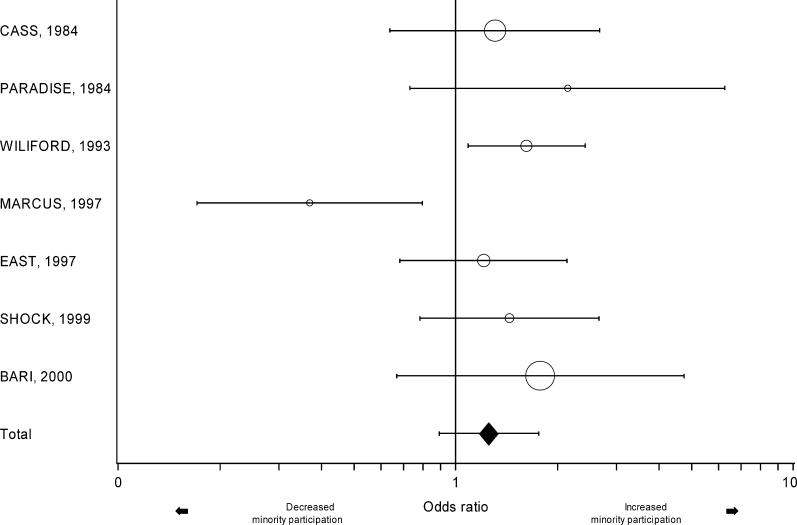
Comparison of Minority versus non-Hispanic White Consent Rates in Surgical Intervention Trials Circle diameter is proportional to the sample size of the individual studies. The diamond represents the overall OR. The vertical line indicates the 95% confidence interval on the OR.

Table 3 reports the consent rates for the seven surgical intervention studies, which categorized all minority groups together. For these seven trials, minorities as a group had a nonsignificantly higher overall consent rate than non-Hispanic whites (65.8% versus 47.8%; OR = 1.26; 95% CI 0.89–1.77; Figure 4). While the test of homogeneity was only nominally significant (*p* = 0.046), six of the seven ORs were greater than one.

**Table 3:**
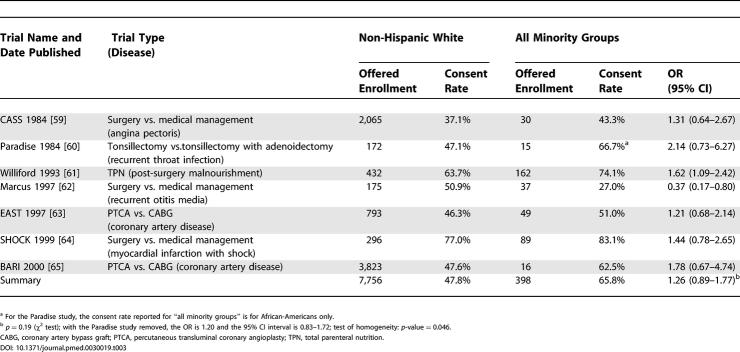
Surgical Intervention Trials

Importantly, of the 20 studies identified, seven offered enrollment to very few minority individuals. For example, the BARI study of percutaneous transluminal coronary angioplasty versus coronary artery bypass graft for coronary artery disease offered enrollment to 3,832 non-Hispanic whites, but to only 16 individuals from all minority groups combined (Table 3). Similarly, the CASS study of surgery versus medical management for angina pectoris offered enrollment to 2,065 non-Hispanic whites, but to only 30 individuals from all minority groups (Table 3).

## Discussion

We identified 20 health research studies that reported the consent rates by race or ethnicity of over 70,000 individuals, the vast majority of whom were from the US. These 20 studies reveal small differences in the rates at which non-Hispanic whites and minorities agree to participate in health research. Indeed, where there are differences in consent rates, individuals from minority groups tend to be slightly more willing to participate in health research, particularly for clinical and surgical intervention studies. These findings contradict the widely held view that racial and ethnic minority groups in the US are less willing than non-Hispanic whites to participate in health research.

Our findings are striking given that they represent the enrollment decisions of over 70,000 individuals, including over 14,000 individuals who were invited to participate in clinical and surgical intervention trials. Furthermore, although we found only 20 studies that reported consent rates by race or ethnicity, these studies represent a broad range of invasiveness and risk, from in-person interviews and medical chart reviews, to drug treatment and surgical trials. These studies also cover a broad range of conditions, including recurrent throat infection, substance abuse, schizophrenia, HIV infection, cancer, and cardiac diseases.

Studies suggest that various factors, including historic abuses like the Tuskegee study, may have undermined minority groups' trust in medical research, as measured by survey questions and focus groups [33,34]. These factors may have increased individuals' suspicions or decreased their level of trust. However, the present analysis reveals that these factors have not resulted in racial and ethnic minorities in the US being less willing to participate in health research [35].

Although we found only small differences in consent rates by race or ethnicity, we did find substantial differences by race and ethnicity in the number of individuals *invited* to participate. In particular, seven of the 17 clinical and surgical intervention studies offered enrollment to relatively few individuals from minority groups, substantially fewer than one would expect based on the percentage of the population composed of minority groups and the incidence of the diseases being studied. For instance, the CASS study of surgery versus medical management for angina pectoris offered enrollment to a total of 2,095 individuals, 2,065 of whom were non-Hispanic whites and only 30 of whom were from all minority groups combined. Yet, as of 1980, 17% of the U.S. population belonged to a minority group, and the estimated prevalence of angina pectoris is higher in minority groups, especially African-Americans and Hispanics, than in non-Hispanic whites [36]. Recognizing that this rough estimate of minority representation in the US fails to take into account other relevant considerations, US demographics and the prevalence of angina pectoris suggest that the CASS study, which recruited individuals in the late 1970s, should have offered enrollment to approximately 356 individuals from minority groups (17% of 2,095), more than ten times the 30 individuals from minority groups actually offered enrollment [37].

Looking at the number of individuals who participated, one might conclude that these studies support the thesis that minorities are less willing to participate in health research in the US. This conclusion is contradicted by the studies' actual consent rates. In the BARI study, individuals from minority groups agreed to participate at a significantly higher rate than non-Hispanic whites (62.5% versus 47.6%). Similarly, individuals from minority groups agreed to participate at a significantly higher rate than non-Hispanic whites in the CASS study (43.3% versus 37.1%).

We found a significant lack of homogeneity for all pooled statistics, with the exception of Hispanics' and non-Hispanic whites' comparative willingness to enroll in clinical intervention trials. This lack of homogeneity indicates that the relative willingness to enroll of minority groups versus non-Hispanic whites varies significantly within the various groups of studies.

The lack of homogeneity suggests that comparative willingness to enroll in specific studies cannot be inferred simply from the type of study, or the racial or ethnic groups in question. Instead, it appears that individuals from minority groups are more willing to enroll in some studies, and non-Hispanic whites are more willing to enroll in others. This finding suggests that willingness to enroll often is more a function of the characteristics of individual studies than a function of racial or ethnic identity. Hence, in cases where a study has difficulty enrolling individuals from a particular group, whether a minority group or non-Hispanic whites, it will be important to assess whether particular characteristics of the study account for this difference. For example, choice of study site may have an important impact on which groups are likely to enroll. Also, the lack of homogeneity suggests that it may be important to conduct further research to determine which characteristics of studies influence the willingness of racial and ethnic groups to participate.

Numerous writers have emphasized the need to increase minority participation in health research [38–42]. Such efforts are important for reasons of justice, and to ensure research findings are generalizable to the entire population. These efforts are especially important given data that minority groups are not represented adequately in some clinical trials [1–4,25]. If efforts to increase minority participation are to succeed, it is vital to understand why minority groups are underrepresented in some research trials. Widespread discussion of past abuses, and racial and ethnic minorities' presumed unwillingness to participate, has focused attention on the attitudes of individuals from minority groups. However, the current data suggest individuals from minority groups, at least in the US, are as willing as non-Hispanic whites to participate in health research when eligible and invited to participate.

This finding suggests that any underrepresentation of minority groups in health research, when it occurs, is likely the result of other factors, such as the fact that some studies invite comparatively few individuals from minority groups to participate [43]. Consequently, efforts to increase minority participation in health research should focus on increasing minority access to research participation, not changing minority attitudes [44–47]. To be successful, these efforts should take into account a number of considerations [48]. Informing minority groups of specific trials and inviting them to participate is an obvious step. In addition, health research trials should try to include sites that are accessible to minority groups, and identify and attempt to address factors that may undermine minority groups' participation in particular, such as the need for child care and reimbursement for travel expenses. Language barriers also may pose difficulties with recruiting some minority groups [49].

Several limitations suggest the need for future research. First, the current findings are limited to published articles that documented consent rates by race or ethnicity. Second, the vast majority of the over 70,000 individuals in the present analysis were from the US. The willingness of minority groups from other countries to participate in health research may differ from the willingness of minority groups in the US. For example, time and cost constraints may preferentially reduce the willingness of individuals from minority groups to participate in health research in general. Yet, this factor may be outweighed in the US by the fact that health care is not guaranteed, and individuals from minority groups may be more likely to use participation in research as a way to obtain access to physicians and health care.

Third, we did not assess minority groups' attitudes toward health research. The current findings do not rule out the possibility that past abuses have resulted in individuals from minority groups being more distrustful of health research than non-Hispanic whites. It may be that past abuses have led to greater distrust among minority groups, but that other factors result in individuals from minority groups being equally willing to participate overall. For instance, some minority groups are more likely to be from lower socioeconomic groups, and individuals from lower socioeconomic groups may be comparatively more willing to participate in research for a number of possible reasons, including a stronger sense of social obligation or to gain access to health treatments.

Fourth, our comprehensive search focused on clinical intervention trials, specifically phase I, phase I/II, phase II, and randomized controlled trials. Our search did not include prevention trials and natural history studies. Individuals from minority groups may be less willing than non-Hispanic whites to participate in these types of studies.

It is widely believed that racial and ethnic minorities are less willing to participate in health research. Such claims often focus on the US, where it is believed that minority groups' relative unwillingness to participate in health research traces to historic abuses, especially the notorious Tuskegee Syphilis Study. We found that racial and ethnic minorities in the US, particularly African-Americans and Hispanics, are as willing to participate, and in some instances more willing to participate, in health research than non-Hispanic whites, when eligible and invited to participate. These findings suggest that efforts to remedy any underrepresentation of minority groups in health research should focus on ensuring equal access to health research for all groups, not on changing attitudes. Efforts to increase minority groups' access to clinical research studies should focus on a range of considerations, including inviting minority groups to participate, using sites accessible to minority groups, and identifying and attempting to address factors that may undermine the participation of individuals from minority groups, such as the need for child care or reimbursement of travel expenses.

## Supporting Information

Figure S1 Selection Process of Clinical Intervention Studies Identified by PubMed Search (26 KB DOC).

Table S1 PubMed Search Terms (63 KB DOC).

Table S2 Web of Science Search Terms Citation Search (44 KB DOC).

**Patient Summary**

**Background**

Health research is meant to determine the best strategies for preventing and treating disease and to inform health policy. Approval of new drugs and health guidelines is usually issued at a national level. Many countries have ethnically and racially diverse populations, and we know that health parameters are not the same for the different groups. To make sure that health policies serve a diverse population, it is important that all ethnic and racial groups participate in health research.

**Why Was This Study Done?**

Several studies have found that minority groups, especially in the US, are often underrepresented in research studies. One possible explanation that has been suggested is that because of past abuses (especially of African-Americans in the notorious Tuskegee Syphilis Study), minorities are less willing to participate in medical research. The authors of this study wanted to test whether this was indeed the case.

**What Did the Researchers Do and Find?**

They looked through the health literature in a systematic way to find all recent studies that reported consent rates by race or ethnicity (every participant in health research has to give “informed consent”). They found 20 such studies, 18 of which were conducted primarily or exclusively in the US, covering a broad range of research from interview-based surveys to clinical trials. Taken together, these studies reported the decision of over 70,000 individuals who were invited to participate. The researchers then compared the consent rates (i.e., the proportion who actually agreed to participate and gave consent) among non-Hispanic whites, African-Americans, and Hispanics. They found very small differences in the overall willingness of minorities to participate in health research compared with non-Hispanic whites. However, they did find that many of the studies invited fewer minority individuals than would be representative for the US patient population.

**What Does This Mean?**

These results suggest that racial and ethnic minority groups, at least in the US, are as willing as non-minority individuals to participate in health research, but that they are underrepresented among the invited participants. Efforts to increase minority participation should therefore focus on offering participation to more minority individuals rather than focusing on changing minority attitudes. It will be important to determine why minorities are underrepresented among people invited to participate in health research. Another interesting question not answered by this study is what motivates individuals from the different groups to accept an invitation and participate in health research both in general and in a particular survey or trial.

**Where Can I Find More Information Online?**

Information on the Tuskeege study:

http://www.cdc.gov/nchstp/od/tuskegee/

http://www.pbs.org/newshour/bb/health/may97/tuskegee_5-16.html

http://www.npr.org/programs/morning/features/2002/jul/tuskegee/commentary.html

Office of Minority Health Affairs of the US National Heart, Lung, and Blood Institute:

http://www.nhlbi.nih.gov/about/omha/

Pages on minority resources and initiatives at the US National Human Genome Research Institute:

http://www.genome.gov/10011199

Report on Inclusion of Women and Minorities in Research from the Office for Protection from Research Risks:

http://www.hhs.gov/ohrp/humansubjects/guidance/hsdc94-01.htm

## Abbreviations

CI: confidence interval
NCHS: National Center for Health Statistics
NHANES: National Health and Nutrition Examination Survey
NHIS: National Health Interview Survey
NIS: National Immunization Survey
OR: odds ratio
