# Supplementary material for: Are Racial and Ethnic Minorities Less Willing to Participate in Health Research?
Source: PLoS Med. 2005 Dec 6;3(2):e19. doi: 10.1371/journal.pmed.0030019 (PMC1298944; doi:10.1371/journal.pmed.0030019)
Supplement: Supplementary file 1 [file pmed.0030019.sg001.doc]

Selection process of clinical intervention studies identified by PubMed search

Studies that included one or more search terms

(n=1681)

Abstracts reviewed to assess whether study might meet inclusion criteria

(n=1106)

Manuscripts reviewed to assess whether study meets inclusion criteria (n=68)

Abstract indicated that the study did not meet inclusion criteria

(n=1038)

Title indicated that the study did not meet inclusion criteria

(n=575)

Studies meeting inclusion criteria

(n=17)

Manuscript indicated that the study did not meet inclusion criteria

(n=51)
