# Supplementary material for: Are Racial and Ethnic Minorities Less Willing to Participate in Health Research?
Source: PLoS Med. 2005 Dec 6;3(2):e19. doi: 10.1371/journal.pmed.0030019 (PMC1298944; doi:10.1371/journal.pmed.0030019)
Supplement: Supplementary file 2 [file pmed.0030019.st001.doc]

**Table S1.** Pubmed Search Terms

| **PUBMED search terms** | **number found** |
| --- | --- |
| **Searching for ANY kind of trial** |  |
| **LIMITS: in title, in English, human** |  |
| selection and bias and study | 24 |
| participation and general* | 36 |
| enroll and refus* | 19 |
| Study and partic* and baseline | 22 |
| Age and refus* | 4 |
| Elderly and refus* | 18 |
| LIMITS: all fields |  |
| generalizable and randomized | 67 |
| enroll and refus* | 19 |
| Generaliz* And enroll* and elig* | 67 |
| Race(title/abstract); English AND Bias (any field)  AND Refus* (any field) | 82 |
| (Refus* AND eligibl*) AND (participat* OR enroll*) | 218 |
| Comprehensive AND cohort AND study | 729 |
| comprehensive AND cohort AND randomized | 53 |
| **LIMITS: clinical trial** |  |
| therapy and refus* and selection and bias | 7 |
| therapy and refus* and enroll | 3 |
| therapy and refus* and registry | 7 |
| enroll* and nonenroll* | 4 |
| Searching for RCTs |  |
| LIMITS: title/abstract, rcts, English, human |  |
| race and refus* | 4 |
| elderly and refus* | 16 |
| age and elig* and refus* | 17 |
| **LIMITS: all fields, rcts, English, human** |  |
| enroll and refus* | 2 |
| therapy and refus* and registry | 5 |
| race and refus* | 10 |
| RCT AND observational | 54 |
| RCT AND parallel | 21 |
| RCT AND concomitant | 13 |
| RCT AND registry | 13 |
| RCT AND refus* | 9 |
| randomization AND non-randomized follow-up | 14 |
| Preference (Title) and RCT | 99 |
| Searching for early phase studies |  |
| Phase I, II, I/II Searches  seeking words “race” “refusal” “enrollment” in titles |  |
| phase I or phase II or phase I/II AND race AND refus* AND eligib* | 2 |
| study and participation and race and refus* | 16 |
| selection and bias and study and race and refus* | 5 |
| [phase I or phase II or phaseI/II] AND participation and race and refus* | 2 |
| **Total** | 1681 |
