# Supplementary material for: Are Racial and Ethnic Minorities Less Willing to Participate in Health Research?
Source: PLoS Med. 2005 Dec 6;3(2):e19. doi: 10.1371/journal.pmed.0030019 (PMC1298944; doi:10.1371/journal.pmed.0030019)
Supplement: Supplementary file 3 [file pmed.0030019.st002.doc]

**Tables S2**. Web of science search terms citation search

| **WEB OF SCIENCE search termS**  **Citation search** | **number found** |
| --- | --- |
| ARTICLE SEARCHES |  |
| Cooper KG 1997 Br J ObGyn | 10 |
| Lambert F 2000 JCE | 4 |
| Smith & Arnesen 1990 | 12 |
| AUTHOR-ARTICLE SEARCHES |  |
| **Olshewski M (author)** | 75 publications |
| Schmoor, Olschewski, Schumacher (1996) | 28 citers |
| Olschewski, Scheurlen (1985) | 19 citers |
| Olschewski, Schumacher, Davis (1992) | 13 citers |
| **Marcus SM** | 99 publications |
| Marcus SM 1990 JCE | 10 citers |
| Stat Med 2001 | 0 citers |
| Cancer 1999 | 7 citers |
| J Clin Epidemiol | 10 citers |
| **Rovers MM** | 20 publications |
| Arch Otolaryngol 2001 | 1 citer |
| Clin Otolaryngol 2001 | 0 citers |
| Arch Dis Child 2001 | 0 citers |
| Ear Hearing 2001 | 8 citers |
| Pediatrics 2000 | 8 citers |
| J Clin Epidemiol 2001 | 2 citers |
| **Albert SM** | 89 publications |
| Neurology 1997 | 18 citers |
| Davis | 43 citers |
| **Total Titles Reviewed** | **371** |
